# NT-proBNP point-of-care testing for predicting mortality in end-stage renal disease: A survival analysis

**DOI:** 10.1016/j.heliyon.2024.e30581

**Published:** 2024-05-03

**Authors:** Chun Chen, Yin-Chen Hsu, Kuang-Wei Chou, Kuo-Song Chang, Ya-Hui Hsu, Wei-Huai Chiu, Chun-Wei Lee, Po-Sheng Yang, Wen-Han Chang, Yao-Kuang Huang, Pang-Yen Chen, Chien-Wei Chen, Yu-Jang Su

**Affiliations:** a Department of Emergency Medicine, Mackay Memorial Hospital, Taipei, Taiwan; b Department of Diagnostic Radiology, Chang Gung Memorial Hospital Chiayi Branch, Chiayi, Taiwan; c Chang Gung University, College of Medicine, Taoyuan, Taiwan; d Department of Nursing, Yuanpei University of Medical Technology, Hsinchu, Taiwan; e Mackay Junior College of Medicine Nursing and Management, Taipei, Taiwan; f Graduate Institute of Automation and Control, National Taiwan University of Science and Technology, Taipei, Taiwan; g Department of Medicine, MacKay Medical College, New Taipei, Taiwan; h Cardiovascular Division, Department of Internal Medicine, MacKay Memorial Hospital, Taipei, Taiwan; i Institute of Public Health, National Yang Ming Chiao Tung University, Taipei, Taiwan; j Department of general surgery, MacKay Memorial Hospital, Taipei, Taiwan; k Graduate Institute of Injury Prevention and Control, Taipei Medical University, Taipei, Taiwan; l Institute of Mechatronic Engineering, National Taipei University of Technology, Taipei, Taiwan; m School of Medicine, Taipei Medical University, Taipei, Taiwan; n Division of Thoracic and Cardiovascular Surgery, Chang Gung Memorial Hospital Chiayi Branch, Chiayi, Taiwan; o Division of Thoracic and Cardiovascular Surgery, Chia Yi Hospital, Ministry of Health and Welfare, Chiayi, Taiwan; p Division of Toxicology, Mackay Memorial Hospital, Taipei, 10449, Taiwan

**Keywords:** N-Terminal-pro brain natriuretic peptide, NT-Pro BNP, End-stage renal disease, ESRD, Survival analysis

## Abstract

This study examines the predictive value of elevated N-terminal-pro brain natriuretic peptide (NT-pro BNP) levels for mortality among patients with end-stage renal disease (ESRD). Data from 768 ESRD patients, excluding those with cancer or lost follow-up, were analyzed using Kaplan–Meier curves and Cox proportional hazards models over three years. Results indicated that patients with very high NT-pro BNP levels had shorter average survival times and a significantly higher risk of mortality (hazard ratio 1.43). Advanced age, ICU admission, and comorbidities like cerebrovascular diseases and chronic obstructive pulmonary disease also contributed to increased mortality risks. Thus, elevated NT-pro BNP is an independent risk factor for mortality in ESRD patients.

## Highlights

• High NT-pro BNP levels, advanced age, and prolonged hospital stay increase mortality risk in end-stage renal disease (ESRD) patients.
• Very high serum NT-pro BNP concentrations reduce 3-year survival in ESRD patients.
• Elevated serum NT-pro BNP levels independently predict mortality (Hazard Ratio 1.43, 95% Confidence Interval: 1.06-1.92).
• Advanced age, intensive care unit (ICU) admission, cerebrovascular accident (CVA), and chronic obstructive pulmonary disease (COPD) significantly increase mortality risk in ESRD patients.

## 1 Introduction

Pre-pro-brain natriuretic peptide (BNP) is synthesized primarily in response to ventricular myocardium stretching and is composed of 134 amino acids. Removing its 26-amino-acid signal peptide from pre-pro-BNP produces a 108-amino acid prohormone known as pro-BNP or BNP 1–108. When secreted, pro-BNP undergoes cleavage by the endo-protease furin, resulting in the biologically active 32-amino acid hormone, BNP, and its inactive counterpart, the 76-amino acid fragment known as N-terminal-pro BNP (NT-pro BNP) [1,2].

The natriuretic peptide clearance receptor is mainly located in the liver, lungs, kidneys, and vascular endothelium. It metabolizes BNP by proteolytic enzymes, such as neutral endopeptidase 24.11 (E−24.11) [1,3]. Compared to BNP, NT-pro BNP seems relatively resistant to neutral endopeptidase degradation [4], and the kidneys primarily extract NT-pro BNP, showing a stronger correlation with the estimated glomerular filtration rate (eGFR) than BNP [5]. Therefore, the plasma concentrations of BNP and NT-pro BNP were influenced by cardiac and renal function and were increased in patients with cardiac and renal impairments.

In addition, BNP and NT-pro BNP are regulated by several physiological factors, such as age, sex, and body mass index (BMI). Studies have shown that women and older people have higher concentrations of NT-pro BNP [2,6,7]. Lower BNP and NT-pro BNP levels are associated with higher BMI values [8,9]. Patients with obesity who underwent gastric surgery showed significantly increased BNP and NT-proBNP levels. Lifestyle changes-induced body weight loss lowers the risk of coronary heart disease (CHD) and increases BNP levels [10].

BNP and NT-pro BNP play essential roles in detecting left ventricular dysfunction, screening for heart disease, and predicting death and cardiovascular (CV) morbidity in patients with heart failure [11]. Both biomarkers are increased in patients with heart failure or renal failure and are therefore described as cardiorenal markers. Although the half-life of BNP is only 20 min, NT-pro BNP has a longer half-life of about 120 min. Hence, NT-pro BNP is believed to be more dependent on renal clearance, has higher circulating levels in the plasma, and is a more stable biomarker than BNP.

Regarding the pathophysiological mechanisms related to NT-proBNP values and renal dysfunction, we summarize the following points.

1. Excretion route: The kidney plays a crucial role in the clearance of NT-proBNP, a biomarker associated with heart failure. As kidney function declines, the clearance of NT-proBNP decreases, leading to elevated concentrations in the bloodstream [12]. NT-proBNP clearance is significantly impacted by kidney function, as evidenced by studies showing that in CKD patients, estimated glomerular filtration rate (eGFR) and hemodynamics are independent predictors of log NT-proBNP [13]. Furthermore, in healthy individuals, the kidneys extract both BNP and NT-proBNP similarly [14].
2. Cardiorenal Interaction: The pathophysiological mechanism underlying cardiorenal syndrome involves a complex interplay between the heart and kidneys. Insufficient cardiac output leads to inadequate renal perfusion, further compromising cardiac function. This results in increased cardiac workload and the release of more NT-proBNP, reflecting the strain on the heart [15]. Pfister et al. reported that NT-pro-BNP, rather than BNP, was the strongest independent predictor of worsening renal function in patients with chronic heart failure [16].
3. Hemodialysis: In the absence of congestive failure or acute myocardial infarction, BNP and NT-pro BNP levels are commonly observed in patients with chronic renal failure (CRF) undergoing hemodialysis. Elevated BNP and NT-pro BNP levels result from extracellular volume overload in patients undergoing hemodialysis with end-stage renal disease (ESRD), causing myocardial stretching and increased left ventricular pressure [17]. Moreover, the dialysis process influences the levels of NT-pro BNP by membrane characteristics, dialysis fluid, ultrafiltration profile, or convective components [2]. The stress on the heart caused by hemodialysis in patients with end-stage renal disease (ESRD) can lead to a significant increase in plasma levels of NT-proBNP [18]. The chronic inflammatory state that may accompany end-stage renal disease (ESRD) and hemodialysis can impact cardiac function, indirectly promoting the release of NT-proBNP [19]. Oxidative stress, which is common in ESRD, contributes to kidney damage progression by promoting renal ischemia, glomerular injury, cell death, and inflammation, potentially affecting cardiac function and NT-proBNP release [19]. NT-proBNP levels are markedly elevated in ESRD patients undergoing hemodialysis, highlighting a potential link between hemodialysis, cardiac strain, and NT-proBNP release. The exact reasons for this elevation are not fully understood, emphasizing the importance of considering the impact of hemodialysis on cardiac function and NT-proBNP levels in ESRD patients.
4. Diagnosis and Monitoring: A systematic review highlighted the importance of NT-proBNP levels in patients with renal impairment and the need for caution when interpreting these levels due to the impact of compromised kidney function on NT-proBNP clearance, potentially leading to misinterpretation of results. In patients with renal dysfunction, the area under the curve (AUC) for NT-proBNP ranged from 0.66 to 0.89 with a median cut-point of 1980 pg/mL, while in patients with preserved renal function, the AUC ranged from 0.72 to 0.95 with a cut-point of 450 pg/mL [20].

In conclusion, elevated NT-proBNP levels serve as not only a marker of heart disease but also a reflection of renal dysfunction. Understanding the intricate relationship between NT-proBNP and renal function is crucial for accurately interpreting NT-proBNP levels, particularly in patients with compromised kidney function. An accurate predictor of mortality in patients with ESRD can help in clinical therapeutic intervention.

We conducted a study that hypothesized that blood NT-pro BNP levels are related to the long-term survival of patients with ESRD. We first aimed to analyze the prognostic factors for mortality due to ESRD. Second, we attempted to determine the correlation between NT-pro BNP and ESRD patients' prognosis. Further understanding of the association of NT-pro BNP with the prognosis of patients with ESRD will help predict the survival and outcome of patients with ESRD and facilitate better clinical decision-making.

## 2 Materials and methods

### 2.1 Study design and participants

**Fig. 1:**
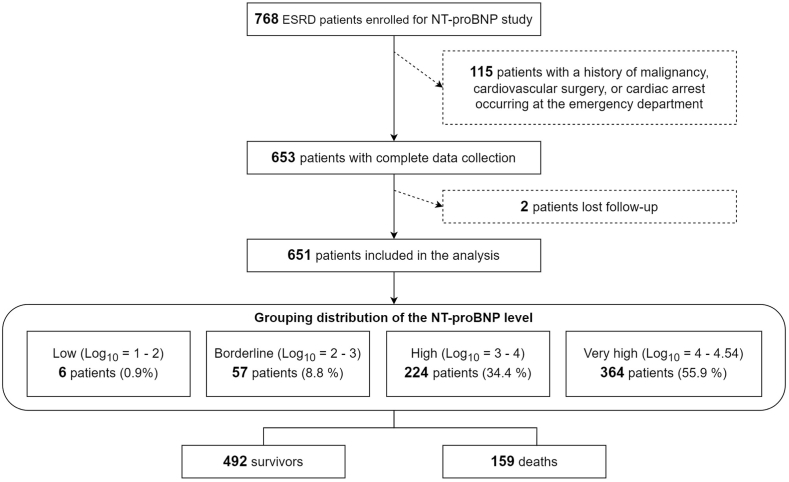
The flow diagram of the patients with ESRD included in this study.

The Institutional Review Board of MacKay Memorial Hospital approved this study under the reference 20MMHIS275e. Between January 1, 2019, and March 31, 2021, our controlled investigation gathered data from ESRD patients who sought treatment at MacKay Memorial Hospital. During this period, 14,381 non-traumatic patients presented to the emergency department. Of this cohort, 13,613 were without ESRD. We have enrolled a total of 768 patients with ESRD but subsequently excluded 115 patients who have a history of malignancy, cardiovascular surgery, or cardiac arrest occurring at the emergency department. [refer to Fig. 1].

This observational and longitudinal analysis focused on a specific population, collecting data on fundamental patient details, demographic attributes, and NT-pro BNP levels. We conducted a longitudinal assessment of mortality based on baseline data. Following this initial evaluation, participants underwent follow-ups every six months for three years. The study tracked mortality until its 1,080th day, using all-cause mortality as the primary outcome. Before participating in any study-related evaluations, all participants furnished written informed consent.

### 2.2 Patients

This analysis focused on 651 ESRD patients with comprehensive clinical and NT-pro BNP level data. We sourced participants from the hospital's outpatient clinics and emergency department. Eligible participants were aged between 20 and 100 years and expressed a willingness to partake in follow-up sessions. However, those with a documented history of malignant diseases were excluded.

### 2.3 Inclusion criteria

● ESRD patients who visited the emergency department.

### 2.4 Exclusion criteria

● history of malignancy
● history of cardiovascular surgery
● cardiac arrest occurring at the emergency department

### 2.5 Measurement of NT-pro BNP levels

Data from point-of-care biomarker testing of the blood samples via the "RADIOMETER" AQT90 FLEX analyzer were collected within 10 min. The detection unit was pg/mL. Taiwan Oriact Co., Ltd. created the AQT90 FLEX analyzer, providing several point-of-care tests in critical care.

### 2.6 Statistical analyses

Data are reported as mean ± standard deviation or number (%) unless otherwise indicated. Survivor and non-survivor descriptors were compared using the two-sample *t*-test for continuous variables and the chi-square test for categorical variables. Kaplan–Meier survival analysis was used to calculate patient survival, and log-rank testing determined discriminative power [21,22]. We used Cox proportional hazard regression to establish the relationship between serum NT-pro BNP level and death, with adjustment for clinical variables [23]. The discriminative power of the Cox regression model was analyzed using C statistics [24]. Multivariate analysis was performed, and hazard ratios for each variable were calculated. In all analyses, P < 0.05 was considered to indicate statistical significance. All P-values were nominal, as no adjustments were made for multiple comparisons.

## 3 Results

### 3.1 Clinical characteristics and NT-pro BNP levels

Of the 768 patients registered at our facility, 653 (85.0 %) had comprehensive clinical and NT-pro BNP level data. During the follow-up, two patients (0.3 %) were unaccounted for, while 159 (20.7 %) passed away. The distribution of baseline serum NT-pro BNP among participants was as follows: low (log_10_ = 1–2, n = 6), borderline (log_10_ = 2–3, n = 57), high (log_10_ = 3–4, n = 224), and very high (log_10_ = 4–4.54, n = 364) [See Fig. 1]. Table 1 contrasts the baseline clinical and physiological features between survivors and non-survivors. Non-survivors tended to be older, had extended hospital stays, more associated conditions, notably chronic obstructive pulmonary disease (COPD) and CV accident (CVA), and elevated serum NT-pro BNP levels. On the other hand, factors like gender, ICU stay duration, and systemic diseases such as diabetes mellitus (DM), myocardial infarction (MI), and liver cirrhosis displayed no marked disparities between the groups.

**Table 1:** Clinical descriptors of the derivation and validation cohorts at the baseline.

	Alive at 3 Years (N = 492)	Dead at 3 Years (N = 159)	P
Demographics			
Male, %	225 (45.73)	81 (50.94)	0.292
Age, years	68.91 (67.59–70.23)	75.21 (73.28–77.14)	<0.001
Hospitalization, days	8.86 (7.77–9.95)	13.15 (10.64–15.66)	<0.001
ICU, day	1.70 (1.24–2.16)	2.35 (1.51–3.19)	0.170
Comorbidities			
COPD, %	19 (3.86)	15 (9.43)	0.011
DM, %	296 (60.16)	85 (53.46)	0.162
MI, %	28 (5.69)	10 (6.29)	0.932
CVA, %	46 (9.35)	28 (17.61)	0.007
Cirrhosis, %	17 (3.46)	10 (6.29)	0.184
Biomarker			
NT-proBNP, mg/dl	15961.83 (14759.11–17164.56)	19741.25 (17562.53–21919.97)	0.003

COPD = chronic obstructive pulmonary disease; DM = diabetes mellitus; MI = myocardial infarction; CVA = cerebral vascular accident.

The values are the mean (confidence interval) or n (%).

### 3.2 Survival analysis

**Fig. 2:**
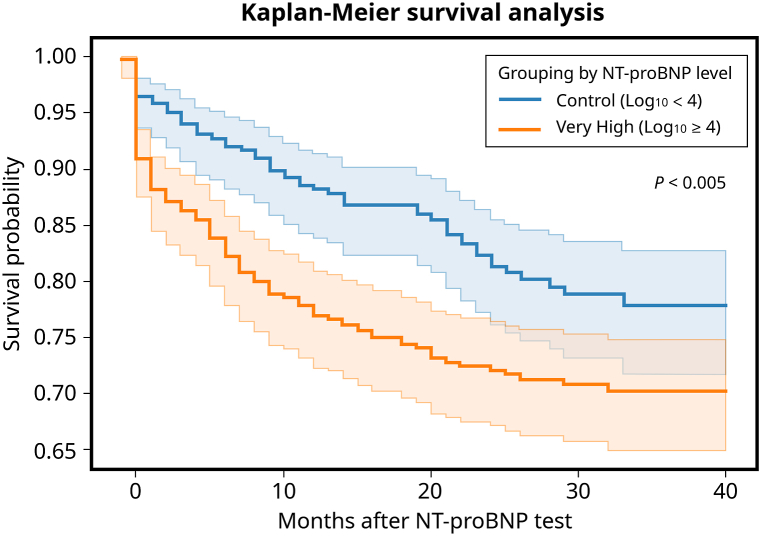
Kaplan–Meier curves illustrate the estimated survival probabilities for two distinct groups based on their serum NT-pro BNP levels. The demarcation is set at log_10_ = 4, which differentiates the high and very high groups. Abbreviations used: COPD denotes chronic obstructive pulmonary disease; DM signifies diabetes mellitus; MI stands for myocardial infarction; and CVA refers to cerebral vascular accident.

NT-pro BNP levels were notably elevated in non-survivors compared to survivors (refer to Table 1). The average survival duration was 25.2 months (95 % confidence interval [CI]: 24.0–26.5 months) in the very high NT-pro BNP group, while it was 23.5 months (95 % CI: 22.2–24.9 months) for the control group. The Kaplan–Meier survival analysis underscored that those with extremely high serum NT-pro BNP concentrations had a diminished likelihood of survival by the close of the 3 years (P < 0.005 as per the log-rank test) [See Fig. 2].

**Fig. 3:**
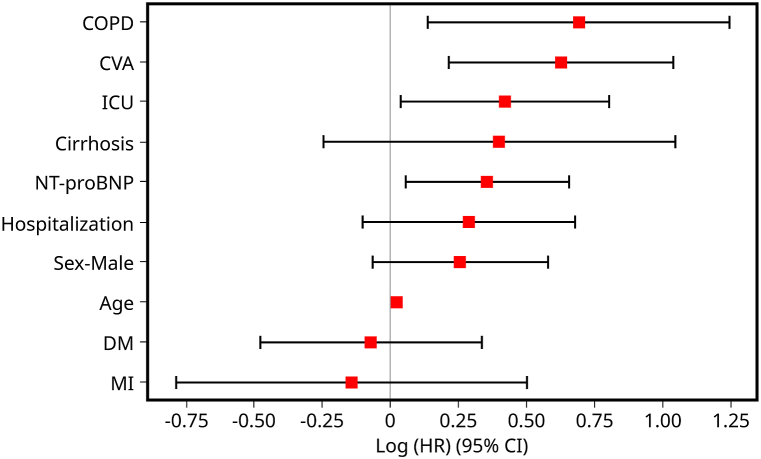
The coefficient plot displays the ranking of variables. It presents hazard ratios (HR) with their respective 95 % confidence intervals (CI) for mortality based on various parameters observed in the study participants. The variables are ranked according to their log (accelerated failure rate).

The Cox regression analysis, detailed in Table 2, revealed that elevated serum NT-pro BNP levels independently associated significantly with an increased mortality risk, showcasing a death hazard ratio of 1.43 (95 % CI: 1.06–1.92; P = 0.02). Factors like age, admission to the ICU, and specific comorbidities such as CVA and COPD had a notable link with heightened mortality. Conversely, a history of DM and MI demonstrated modest survival advantages, although these lacked statistical significance after adjusting for gender, age, hospital stay, and ICU care duration. The C-statistic of the Cox regression model stood at 0.676 [Fig. 3]. graphically presents the variable rankings based on their log (accelerated failure rate).

**Table 2:** Hazard ratios and confidence intervals for death are based on the multivariable Cox proportional hazards model.

Parameter	Increment	Hazard Ratio	95 % Confidence Interval	P-value
Demographics				
Age	1-year increase	1.02	1.01–1.03	<0.005
ICU	Categorical status	1.53	1.04–2.24	0.03
Male	Categorical status	1.29	0.94–1.78	0.12
Hospitalization	Categorical status	1.33	0.90–1.97	0.15
Comorbidities				
CVA	Categorical status	1.87	1.24–2.82	<0.005
COPD	Categorical status	2.00	1.15–3.47	0.01
Cirrhosis	Categorical status	1.49	0.78–2.85	0.23
MI	Categorical status	0.87	0.45–1.66	0.67
DM	Categorical status	0.93	0.62–1.40	0.73
Biomarker				
NT-proBNP (mg/dl)	1 log increase	1.43	1.06–1.92	0.02

COPD = chronic obstructive pulmonary disease; DM = diabetes mellitus; MI = myocardial infarction; CVA = cerebral vascular accident.

**Fig. 4:**
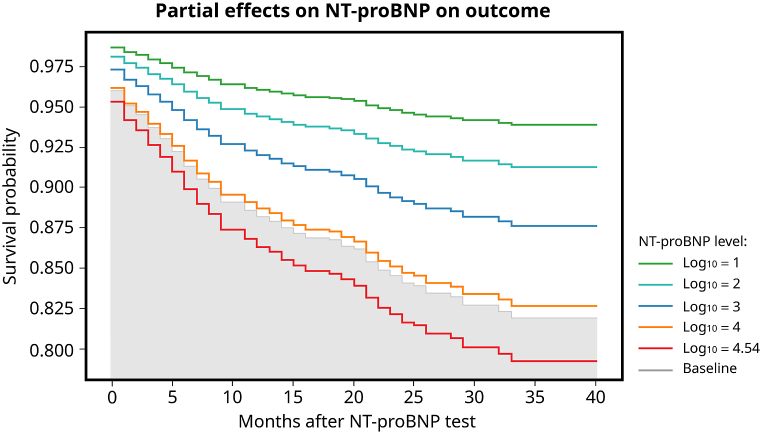
The survival rates differ across various serum NT-pro BNP level thresholds (log_10_ = 1, 2, 3, 4, and 4.54) when compared to baseline measurements. The graphical representation reveals a tiered trend, emphasizing that at any specific time point, patients with lower NT-pro BNP levels generally have a higher likelihood of survival compared to those with extremely elevated serum NT-pro BNP levels.

[Fig. 4] illustrates the survival differentiation based on various thresholds of serum NT-pro BNP concentrations (log_10_ = 1, 2, 3, 4, and 4.54) relative to their foundational performance. The graph depicted a layered pattern, emphasizing that, at any particular point, patients boasted superior survival chances compared to those with highly elevated serum NT-pro BNP levels.

## 4 Discussion

Our study revealed that demographic factors, such as age and days of hospitalization, were significantly associated with increased mortality (P < 0.001). This result is reasonable because these factors represent more complicated and severe conditions. Comorbidities, such as CVA and COPD, were also associated with increased mortality. Decreased renal function is associated with an increased risk of ischemic or hemorrhagic stroke, and patients face a significantly higher risk of post-stroke mortality [[25], [26], [27]]. A strong association between COPD and chronic kidney disease (CKD) has emerged over the past decade [28]. There is a 2.2-fold prevalence of CKD in patients with COPD compared to those with no COPD. Studies from many countries have suggested that COPD increases mortality risk among patients with advanced CKD [[29], [30], [31], [32]]. Our study further confirmed the findings of previous studies.

Although the mean values of NT-pro BNP in both groups were very high, we found that NT-pro BNP levels were significantly higher in non-survivors. Plasma NT-pro BNP concentrations <400 pg/mL are associated with a minimal likelihood of heart failure [33]. However, the best cut-off values for detecting heart failure in patients with ESRD may be needed to predict prognosis and decide on medical intervention. Fluid overload in hemodialysis patients is multifactorial and includes excessive water intake and inadequate dialysis. This fluid imbalance status may contribute to elevated levels of plasma NT-pro BNP without cardiac dysfunction [34]. Even in asymptomatic and euvolemic patients with ESRD, the plasma concentration of NT-pro BNP may still be greater than 20 times the upper limit [3].

While the diagnostic precision of NT-pro BNP for heart failure diminishes in ESRD patients, numerous studies suggest its promise as a cardiac biomarker for mortality prediction in those undergoing dialysis. Schaub et al. conducted a systematic review and meta-analysis focused on patients with eGFR <60 mL/min/1.73 m^2^ and found that elevated NT-pro BNP concentrations correlated with an increased risk of all-cause mortality [20]. In another systematic review and meta-analysis targeting ESRD patients, Harrison et al. unveiled heightened risks for cardiovascular (CV) and all-cause mortality with rising NT-pro BNP levels [35]. Apple et al. determined that surges in plasma NT-pro BNP, high-sensitivity C-reactive protein (hs-CRP), cardiac troponin T (cTnT), and cardiac troponin I (cTnI) were all strong predictors of mortality among ESRD patients. Additionally, NT-pro BNP outperformed hs-CRP and cTnT regarding the area under the receiver operating characteristic curve for predicting mortality [36].

In the realm of peritoneal dialysis (PD), Wang AY and colleagues demonstrated that NT-pro BNP concentrations serve as crucial predictors for mortality and unfavorable cardiovascular incidents [37]. Beyond cardiovascular consequences, some research efforts indicate a link between elevated NT-pro BNP levels and a heightened risk of rapid transition from CKD to ESRD [16,38].

The present study used point-of-care testing (POCT) for NT-pro BNP, and the results revealed that the levels of POCT NT-pro BNP were significantly higher in non-survivors than survivors. POCT NT-pro BNP level is significantly associated with mortality risk in patients with ESRD. In addition, we obtained the NT-pro BNP values via POCT within 10 min. So, it may be possible to differentiate and transfer patients to the next suitable therapeutic unit. Compared to traditional laboratory methods, POCT shortens the examination time. In addition, samples can be collected at the bedside (blood from fingertips). Therefore, it is more convenient and time-saving for doctors to predict prognosis and make decisions.

Fig. 2 demonstrates that in the very high NT-pro BNP group (log_10_ ≥ 4, 10000), the survival rate decreased within the following three years. The mean survival time was 23.5 months (95 % CI: 22.2–24.9 months) in the very high NT-pro BNP group and 25.2 months (95 % CI: 24.0–26.5 months) in the NT-pro BNP log_10_ <4 group, and the difference was statistically significant. Although 10,000 may not be a precise cut-off point for poor prognosis, the result proves that higher NT-pro BNP levels indicate higher long-term mortality. In addition, Fig. 4 shows that survival varies for different thresholds of serum NT-pro BNP, highlighting that patients have higher survival probabilities at any given time than those with very high serum NT-pro BNP levels. The figure also shows that the survival rates progressively decreased in the next 40 months with a more significant NT-pro BNP level threshold, allowing us to predict the patient's prognosis based on the POCT NT-pro BNP value.

Harrison's systematic review and meta-analysis on ESRD patients delved into the nuanced relationship between specific NT-pro BNP thresholds and their associated hazard ratios [35]. As the thresholds of NT-pro BNP increased from >2000 pg/mL to >15,000 pg/mL, the hazard ratios for cardiovascular mortality also progressively heightened. Similarly, the risk for all-cause mortality amplified considerably across all NT-pro BNP thresholds, beginning from >1000 pg/mL and escalating to >20,000 pg/mL. Notably, the review underscored that an NT-pro BNP level exceeding 10,000 pg/mL correlated with a quadrupled risk of CV mortality, alongside substantial risks for both all-cause mortality and CV events. In Fig. 2, the "very high" category for NT-pro BNP was characterized by a log_10_ range of 4–4.54. That equals a plasma NT-pro BNP concentration from 10,000 to 35,000 pg/mL. It was observed that patients falling within this range exhibited diminished survival rates.

One can reasonably infer that among ESRD patients, those with elevated NT-pro BNP might be experiencing severe medical complications like advanced heart disease, sepsis, or a state of shock, thereby leading to an elevated mortality rate [39,40]. Fig. 4 also illustrates that patients with an NT-pro BNP level of log_10_ = 4.54 have an even lower survival rate than those at log_10_ = 4. This pattern implies a possible correlation between escalating NT-pro BNP levels and increasing mortality, especially among those with exceedingly high NT-pro BNP concentrations.

The reportable spectrum for the AQT90 FLEX NT-pro BNP test ranges from 70 to 35,000 ng/L. Readings that surpass the upper boundary or fall beneath the lower one are denoted using ">" and "<" symbols, respectively. This range information is derived from the AQT90 FLEX NT-pro BNP test kit's package insert by Radiometer Medical [41]. Historically, when the Point-of-Care Testing (POCT) methodology was being developed, NT-pro BNP concentrations exceeding 35,000 pg/mL were deemed to offer limited clinical diagnostic utility. Hence, the set upper threshold for POCT NT-pro BNP stood at 35,000 pg/mL. As for the conjecture that NT-pro BNP values exceeding 35,000 pg/mL might correlate with an even graver prognosis, it necessitates more comprehensive data and subsequent analysis.

Elevated serum NT-pro BNP levels have been consistently associated with adverse outcomes in various medical conditions. Higher NT-pro BNP levels were linked to increased mortality in patients with acute ischemic stroke [42]. Similarly, elevated NT-pro BNP levels were observed to correlate with postoperative outcomes in older patients undergoing transcatheter aortic valve replacement [43]. These findings align with the results of the current study, where non-survivors exhibited significantly higher NT-pro BNP levels compared to survivors, indicating a strong association between elevated NT-pro BNP and mortality risk [44].

Moreover, the study highlighted the predictive value of NT-pro BNP in sepsis-induced myocardial dysfunction, further emphasizing the significance of NT-pro BNP as a predictor of adverse cardiac events [45]. Additionally, the research demonstrated the importance of NT-pro BNP levels in acute exacerbation of COPD patients, showing that elevated NT-pro BNP was associated with poor outcomes [46].

Furthermore, the study emphasized the role of NT-pro BNP in assessing volume status and cardiac function in hemodialysis patients, indicating the utility of NT-pro BNP as a marker of cardiac abnormalities in specific patient populations [47]. These diverse studies collectively underscore the consistent relationship between elevated NT-pro BNP levels and adverse clinical outcomes across various medical conditions, reinforcing the value of NT-pro BNP as a prognostic indicator in cardiovascular and systemic diseases.

The study's findings on the association between NT-pro BNP levels and patient mortality risk have significant practical implications for actual clinical practice. Healthcare providers can leverage this information to enhance patient care and outcomes by highlighting the relevance of NT-pro BNP as a prognostic marker.

Firstly, the study's identification of elevated NT-pro BNP levels in non-survivors compared to survivors underscores the importance of monitoring NT-pro BNP levels in clinical practice. Regular assessment of NT-pro BNP levels can aid in risk stratification and early identification of patients at higher mortality risk, allowing for timely interventions and personalized management strategies.

Secondly, the study's survival analysis, demonstrating a diminished likelihood of survival in patients with extremely high serum NT-pro BNP concentrations, emphasizes the need for close monitoring and proactive management of patients with elevated NT-pro BNP levels. Healthcare providers can use this information to prioritize care for high-risk patients and tailor treatment plans to address the underlying factors contributing to elevated NT-pro BNP levels.

Moreover, the Cox regression analysis revealing a significant association between elevated NT-pro BNP levels and increased mortality risk provides valuable insights for prognostication in clinical practice. Integrating NT-pro BNP levels into risk assessment models can enhance the accuracy of mortality predictions and guide decision-making regarding treatment strategies and follow-up care.

In actual clinical settings, the study's findings can inform healthcare professionals about the importance of considering NT-pro BNP levels as part of routine assessments for patients, particularly those at higher risk of adverse outcomes. By incorporating NT-pro BNP monitoring into clinical practice, healthcare providers can improve risk stratification, enhance patient outcomes, and optimize care delivery for individuals with elevated mortality risk.

This study has some limitations that need to be considered. First, there are some noncardiac comorbidities and critical illnesses that can trigger an increase in NT-proBNP levels. However, this study only focused on the fundamental items. Second, the study cohort was recruited based on a review of the medical records at a point in time. Any diagnosis made after the patient leaves the emergency department may not have been considered in this study and is therefore limited to some extent. Finally, this study was conducted in a single medical institute. A large-scale, prospective study is required to explore further the benefits of using POCT NT-pro BNP in assessing the prognosis of the ESRD population.

## 5 Conclusion

Patients with ESRD typically exhibit higher NT-proBNP levels compared to the general population. Nonetheless, this elevation doesn't diminish NT-proBNP's value as a prognostic marker. Elevated serum NT-pro BNP levels are significantly and independently linked to an increased mortality risk, with a death hazard ratio of 1.43 (95 % CI: 1.06–1.92). Compared to those with highly elevated POCT NT-pro BNP concentrations, patients generally exhibited better survival probabilities at any given time. A clear correlation exists between the values of POCT NT-pro BNP and mortality rates. Factors like advanced age, ICU admissions, and comorbid conditions such as CV and COPD significantly heighten mortality risks among ESRD patients. In essence, a high POCT NT-pro BNP reading signals a grim prognosis, underscoring the need for physicians to undertake more rigorous clinical interventions. In short, patients with ESRD have higher NT-pro BNP values than the general population, but this does not prevent its role as a patient prognostic indicator. Time-saving Point-Of-Care, Testing NT-pro BNP values correlate with the patient's later mortality rate.

## Funding

This study was funded by 10.13039/100012553Chang Gung Memorial Hospital, Taiwan, under Contract Nos. CMRPG6K0341, CMRPG6K0342, and CMRPG6L0351.

## Data availability statement

The datasets used in the study are available from the corresponding author upon reasonable request.

## CRediT authorship contribution statement

**Chun Chen:** Writing – original draft. **Yin-Chen Hsu:** Data curation. **Kuang-Wei Chou:** Writing – review & editing. **Kuo-Song Chang:** Writing – review & editing. **Ya-Hui Hsu:** Writing – review & editing. **Wei-Huai Chiu:** Writing – review & editing. **Chun-Wei Lee:** Conceptualization. **Po-Sheng Yang:** Conceptualization. **Wen-Han Chang:** Conceptualization. **Yao-Kuang Huang:** Conceptualization. **Pang-Yen Chen:** Writing – review & editing, Writing – original draft, Data curation, Conceptualization. **Chien-Wei Chen:** Writing – review & editing, Writing – original draft, Data curation, Conceptualization. **Yu-Jang Su:** Writing – review & editing, Investigation, Conceptualization.

## Declaration of competing interest

The authors declare that they have no known competing financial interests or personal relationships that could have appeared to influence the work reported in this paper.
